# Supplementary material for: A One Health Approach Metagenomic Study on Antimicrobial Resistance Traits of Canine Saliva
Source: Antibiotics (Basel). 2025 Apr 25;14(5):433. doi: 10.3390/antibiotics14050433 (PMC12108403; doi:10.3390/antibiotics14050433)

antibiotics

Article

A One Health Approach Metagenomic Study on Antimicrobial Resistance Traits of Canine Saliva

Adrienn Gréta Tóth [1,2], Darinka Lilla Tóth [3], Laura Remport [4], Imre Tóth [5,6], Tibor Németh [7], Attila Dubecz [8], Árpád V. Patai [9], Zsombor Wagenhoffer [2], László Makrai [10] and Norbert Solymosi [1,11,*]

[1] Centre for Bioinformatics, University of Veterinary Medicine, 1078 Budapest, Hungary; toth.adrienn.greta@univet.hu
[2] Institute for Animal Breeding, Nutrition and Laboratory Animal Science , University of Veterinary Medicine, 1078 Budapest, Hungary; wagenhoffer.zsombor@univet.hu
[3] School of Biodiversity, One Health & Veterinary Medicine, University of Glasglow, Glasgow G12 8QQ, UK; darinka.lilla.toth@gmail.com
[4] OnlyVet Veterinary Referral Center, 69800 Saint-Priest, France; remportlaura@gmail.com
[5] Department of Operative Tecniques and Surgical Research, Faculty of Medicine, University of Debrecen, 4032 Debrecen, Hungary; drtothimre@bazmkorhaz.hu
[6] Department of Thoracic Surgery, Borsod-Abaúj-Zemplén County Hospital, and University Teaching Hospital, 3526 Miskolc, Hungary
[7] Department and Clinic of Surgery and Ophthalmology, University of Veterinary Medicine, 1078 Budapest, Hungary
[8] Department of Surgery, Paracelsus Medical University, 90419 Nuremberg, Germany; attila.dubecz@klinikum-nuernberg.de
[9] Division of Interventional Gastroenterology, Department of Surgery, Transplantation and Gastroenterology, Semmelweis University, 1082 Budapest, Hungary; patai.arpad@semmelweis.hu
[10] Autovakcina Ltd., 1171 Budapest, Hungary; autovakcina@gmail.com
[11] Department of Physics of Complex Systems, Eötvös Loránd University, 1117 Budapest, Hungary
* Correspondence: solymosi.norbert@gmail.com; Tel.: +36-30-9347-069

check for updates

Academic Editor: Piera Anna Martino

Received: 20 March 2025
Revised: 10 April 2025
Accepted: 15 April 2025
Published: 25 April 2025

Citation: Tóth, A. G.; Tóth, D. L.; Remport, L.; Tóth, I.; Németh, T.; Dubecz, A.; Patai, Á. V.; Wagenhoffer, Z.; Makrai, L.; Solymosi, N. A One Health Approach Metagenomic Study on Antimicrobial Resistance Traits of Canine Saliva. *Antibiotics* **2025**, *14*, 433. https://doi.org/10.3390/antibiotics14050433

Copyright: © 2025 by the authors. Licensee MDPI, Basel, Switzerland. This article is an open access article distributed under the terms and conditions of the Creative Commons Attribution (CC BY) license (https://creativecommons.org/licenses/by/4.0/).

Table S1. The number of samples containing ARGs (ARG+) and lacking ARGs (ARG-) associated with the questions regarding certain physical traits of dogs involved in the survey. "Group 1" and "Group 2" represent the order of traits indicated in the "Trait" column of the table. In column "Approach", "A" indicates ARGs detected in any canine metagenomic samples, "B" is for higher-public-health-risk ARGs detected in any canine metagenomic samples, and "C" stands for higher-public-health-risk ARGs detected in ESKAPE pathogens. No stars is: *p*-value > 0.05 (not statistically significant), one star (*): *p*-value ≤ 0.05 (statistically significant at the 5% level), two stars (**): *p*-value ≤ 0.01 (statistically significant at the 1% level).

Trait<br>Group 1 / Group 2	Approach	Number of samples<br>Group 1<br>ARG+	 <br>ARG-	 Group 2<br>ARG+	 <br>ARG-	OR (95%CI)	p-value
**When the dog is standing next to someone of average height, how high are his shoulders?**							
Under knee / knee high and above	A	503	37	1035	95	1.25 (0.83 - 1.91)	0.288
	B	329	212	609	521	1.33 (1.07 - 1.65)	0.008**
	C	15	526	45	1085	0.69 (0.35 - 1.27)	0.261
**What color is the dog?**							
Whites / other colors	A	795	54	685	70	1.50 (1.02 - 2.22)	0.031*
	B	496	353	405	351	1.22 (0.99 - 1.49)	0.056
	C	36	813	21	735	1.55 (0.87 - 2.82)	0.137
Blacks / other colors	A	149	11	1331	113	1.15 (0.60 - 2.42)	0.757
	B	90	71	811	633	0.99 (0.70 - 1.39)	1.000
	C	5	156	52	1392	0.86 (0.26 - 2.18)	1.000
Blacks / whites	A	149	11	795	54	0.92 (0.46 - 2.00)	0.860
	B	90	71	496	353	0.90 (0.63 - 1.29)	0.601
	C	5	156	36	813	0.72 (0.22 - 1.89)	0.664
Whites / blacks, browns, reds, yellows	A	795	54	543	58	1.57 (1.05 - 2.36)	0.022*
	B	496	353	324	278	1.21 (0.97 - 1.50)	0.086
	C	36	813	18	584	1.44 (0.79 - 2.71)	0.260
Whites, merles / blacks, browns, reds, yellows	A	816	55	543	58	1.58 (1.06 - 2.37)	0.022*
	B	507	364	324	278	1.19 (0.96 - 1.48)	0.098
	C	36	835	18	584	1.40 (0.76 - 2.64)	0.263
Whites, merles, grays / blacks, browns, reds, yellows	A	865	56	543	58	1.65 (1.10 - 2.47)	0.012*
	B	531	390	324	278	1.17 (0.94 - 1.44)	0.154
	C	36	885	18	584	1.32 (0.72 - 2.49)	0.396
**How much white fur does dog have?**							
All whites / others	A	194	25	1341	107	0.62 (0.39 - 1.03)	0.044*
	B	130	89	806	643	1.17 (0.86 - 1.57)	0.307
	C	7	212	53	1396	0.87 (0.33 - 1.95)	0.847
Almost all whites / others	A	310	33	1225	99	0.76 (0.50 - 1.19)	0.216
	B	208	135	728	597	1.26 (0.99 - 1.62)	0.059
	C	13	330	47	1278	1.07 (0.53 - 2.04)	0.871
No white / any white	A	670	55	865	77	1.08 (0.75 - 1.59)	0.715
	B	402	324	534	408	0.95 (0.78 - 1.16)	0.619
	C	21	705	39	903	0.69 (0.38 - 1.21)	0.187
All white / no white	A	194	25	670	55	0.64 (0.38 - 1.10)	0.095
	B	130	89	402	324	1.18 (0.86 - 1.62)	0.313
	C	7	212	21	705	1.11 (0.39 - 2.75)	0.821
**Is dog's tail curly?**							
Not curly / curly	A	1036	93	396	32	0.90 (0.57 - 1.38)	0.677
	B	625	505	242	186	0.95 (0.76 - 1.20)	0.689
	C	37	1093	16	412	0.87 (0.47 - 1.70)	0.641
**What is the dog's ear shape like?**							
Long pendant ears / others	A	181	14	1355	118	1.13 (0.63 - 2.17)	0.779
	B	119	76	818	656	1.26 (0.92 - 1.73)	0.146
	C	9	186	50	1424	1.38 (0.59 - 2.89)	0.406
Surgically cropped ears / others	A	18	2	1518	130	0.77 (0.18 - 6.92)	0.669
	B	10	10	927	722	0.78 (0.29 - 2.10)	0.653
	C	1	19	58	1591	1.44 (0.03 - 9.40)	0.515
**Are dog's eyes different colors?**							
Not different eyes / different eyes	A	1476	126	50	3	0.70 (0.14 - 2.23)	0.794
	B	900	703	32	21	0.84 (0.46 - 1.52)	0.576
	C	54	1549	6	47	0.27 (0.11 - 0.82)	0.011*
**How long is dog's fur on his back and sides?**							
Long / short	A	216	13	781	74	1.57 (0.85 - 3.15)	0.170
	B	134	95	479	376	1.11 (0.82 - 1.51)	0.548
	C	15	214	26	829	2.23 (1.08 - 4.47)	0.019*
Long / medium, short	A	216	13	1296	118	1.51 (0.83 - 2.98)	0.189
	B	134	95	787	628	1.13 (0.84 - 1.51)	0.431
	C	15	214	43	1372	2.24 (1.13 - 4.19)	0.012*
**Does the dog have soft fur or rough and bristly fur?**							
Rough(wiry) / soft	A	220	17	1205	109	1.17 (0.68 - 2.12)	0.698
	B	142	95	735	580	1.18 (0.88 - 1.58)	0.256
	C	6	231	51	1264	0.64 (0.22 - 1.52)	0.451

Table S2. The number of samples containing ARGs (ARG+) and lacking ARGs (ARG-) associated with questions regarding the behaviour of dogs involved in the survey. "Group 1" and "Group 2" represent answers "Never" and "Not never" (Always, Often, Sometimes, Rarely), respectively. In column "Approach", "A" indicates ARGs detected in any canine metagenomic samples, "B" is for higher-public-health-risk ARGs detected in any canine metagenomic samples, and "C" stands for higher-public-health-risk ARGs detected in ESKAPE pathogens. No stars is: *p*-value > 0.05 (not statistically significant), one star (*): *p*-value ≤ 0.05 (statistically significant at the 5% level)

Question	Approach	Number of samples				OR (95%CI)	p-value
		Never		Not never			
		ARG+	ARG-	ARG+	ARG-		
Dog walks away or avoids being patted	A	705	50	841	80	1.34 (0.92 - 1.98)	0.120
	B	422	333	521	401	0.98 (0.80 - 1.19)	0.805
	C	36	719	24	898	1.87 (1.08 - 3.31)	0.024*
Dog shows barrier aggression	A	1037	82	507	50	1.25 (0.85 - 1.83)	0.249
	B	634	486	307	250	1.06 (0.86 - 1.31)	0.566
	C	43	1077	17	540	1.27 (0.70 - 2.39)	0.486
Dog lays in one place all day long	A	734	68	815	64	0.85 (0.58 - 1.23)	0.366
	B	434	368	513	367	0.84 (0.69 - 1.03)	0.085
	C	28	774	31	849	0.99 (0.57 - 1.72)	1.000
Dog tilts his head	A	140	20	1384	110	0.56 (0.33 - 0.98)	0.029*
	B	92	68	838	656	1.06 (0.75 - 1.50)	0.801
	C	5	155	53	1441	0.88 (0.27 - 2.23)	1.000

Table S3. The number of samples containing ARGs (ARG+) and lacking ARGs (ARG-) associated with questions regarding the behaviour of dogs involved in the survey. "Group 1" and "Group 2" represent answers "Agree" (Strongly agree, Agree) and "Disagree" (Strongly disagree, disagree), respectively. In column "Approach", "A" indicates ARGs detected in any canine metagenomic samples, "B" is for higher-public-health-risk ARGs detected in any canine metagenomic samples, and "C" stands for higher-public-health-risk ARGs detected in ESKAPE pathogens. No stars is: p-value > 0.05 (not statistically significant), one star (*): p-value $\leq$ 0.05 (statistically significant at the 5% level), two stars (**): p-value $\leq$ 0.01 (statistically significant at the 1% level) and three stars (***): p-value $\leq$ 0.001 (statistically significant at the 0.1% level).

Question	Approach	Number of samples				OR (95%CI)	p-value
		Agree		Disagree			
		ARG+	ARG-	ARG+	ARG-		
Dog wants to play	A	1295	117	93	4	0.48 (0.12 - 1.30)	0.176
	B	779	634	66	31	0.58 (0.36 - 0.91)	0.015*
	C	48	1365	7	90	0.45 (0.20 - 1.22)	0.082
Dog becomes aggressive when	A	262	22	1075	93	1.03 (0.63 - 1.76)	1.000
excited	B	144	140	681	488	0.74 (0.56 - 0.96)	0.023*
	C	7	277	43	1126	0.66 (0.25 - 1.51)	0.369
Dog enjoys playing with toys	A	1228	110	203	11	0.61 (0.29 - 1.15)	0.132
	B	738	601	131	83	0.78 (0.57 - 1.06)	0.103
	C	47	1292	10	204	0.74 (0.36 - 1.67)	0.431
Dog gets bored in play quickly	A	389	27	823	87	1.52 (0.96 - 2.48)	0.072
	B	242	174	491	420	1.19 (0.93 - 1.52)	0.153
	C	13	403	37	874	0.76 (0.37 - 1.49)	0.442
Dog seeks constant activity	A	394	39	880	69	0.79 (0.52 - 1.23)	0.280
	B	208	226	578	371	0.59 (0.47 - 0.75)	0.000***
	C	14	420	37	912	0.82 (0.41 - 1.58)	0.645
Dog may become aggressive if he is	A	197	22	1208	104	0.77 (0.47 - 1.32)	0.289
frustrated with something	B	114	105	748	565	0.82 (0.61 - 1.10)	0.186
	C	6	213	43	1270	0.83 (0.29 - 2.00)	0.836

Dog is relaxed when greeting	A	491	32	882	92	1.60 (1.04 - 2.51)	0.030*
people	B	315	208	519	456	1.33 (1.07 - 1.66)	0.010*
	C	20	503	34	941	1.10 (0.59 - 1.99)	0.772
Dog is boisterous	A	684	63	578	47	0.88 (0.58 - 1.33)	0.551
	B	392	356	365	260	0.78 (0.63 - 0.98)	0.029*
	C	31	717	16	609	1.65 (0.86 - 3.25)	0.136
Dog aggres-sively guards coveted items	A	295	26	1098	95	0.98 (0.62 - 1.61)	0.908
(e.g., stolen item, treats, food bowl)	B	174	147	677	517	0.90 (0.70 - 1.17)	0.447
	C	13	308	42	1152	1.16 (0.56 - 2.23)	0.617
Dog is playful with other dogs	A	946	85	314	22	0.78 (0.46 - 1.28)	0.351
	B	564	468	204	132	0.78 (0.60 - 1.01)	0.058
	C	37	995	14	322	0.86 (0.44 - 1.74)	0.620
Dog likes to chase squirrels,	A	1188	105	227	20	1.00 (0.57 - 1.66)	1.000
birds, or other small animals	B	725	569	143	104	0.93 (0.70 - 1.23)	0.624
	C	37	1257	16	231	0.43 (0.23 - 0.83)	0.007**
Dog is as active as he has been	A	1206	115	248	12	0.51 (0.25 - 0.94)	0.025*
	B	716	606	174	86	0.58 (0.44 - 0.78)	0.000***
	C	46	1276	10	250	0.90 (0.44 - 2.03)	0.716
Dog sleeps more, is less awake	A	509	37	675	58	1.18 (0.76 - 1.87)	0.453
	B	335	211	390	344	1.40 (1.11 - 1.77)	0.004**
	C	19	527	35	699	0.72 (0.38 - 1.31)	0.325

Dog behaves aggressively in	A	286	34	1071	82	0.64 (0.42 - 1.01)	0.046*
response to perceived threats from	B	175	145	656	498	0.92 (0.71 - 1.19)	0.524
people	C	9	311	44	1110	0.73 (0.31 - 1.54)	0.498
Dog behaves fearfully towards	A	413	51	942	68	0.58 (0.39 - 0.87)	0.007**
unfamiliar people	B	249	216	593	417	0.81 (0.65 - 1.02)	0.070
	C	15	450	38	972	0.85 (0.43 - 1.61)	0.655
Dog seems to get excited for no reason	A	291	26	1031	86	0.93 (0.58 - 1.54)	0.812
	B	174	143	651	467	0.87 (0.67 - 1.13)	0.303
	C	11	306	42	1076	0.92 (0.42 - 1.85)	1.000
Dog behaves aggressively	A	192	27	1211	91	0.53 (0.33 - 0.88)	0.009**
towards unfamiliar people	B	126	93	748	555	1.01 (0.75 - 1.36)	1.000
	C	4	215	51	1252	0.46 (0.12 - 1.26)	0.168
Dog chases bicycles, joggers, and	A	311	22	1014	80	1.12 (0.68 - 1.91)	0.717
skateboarders	B	192	141	612	482	1.07 (0.83 - 1.39)	0.614
	C	10	323	40	1054	0.82 (0.36 - 1.68)	0.733
Dog retrieves objects (e.g., balls,	A	961	86	409	29	0.79 (0.49 - 1.24)	0.338
toys, sticks)	B	564	483	275	163	0.69 (0.55 - 0.88)	0.002**
	C	38	1009	15	423	1.06 (0.56 - 2.10)	1.000
Dog behaves aggressively toward	A	322	33	917	78	0.83 (0.53 - 1.31)	0.431
other dogs	B	198	157	557	438	0.99 (0.77 - 1.28)	0.950
	C	6	349	46	949	0.35 (0.12 - 0.84)	0.015*

Dog is dominant over other dogs	A	428	36	696	62	1.06 (0.68 - 1.67)	0.829
	B	265	199	421	337	1.07 (0.84 - 1.36)	0.594
	C	16	448	29	729	0.90 (0.45 - 1.73)	0.876
Dog behaves aggressively towards cats	A	421	38	726	52	0.79 (0.50 - 1.26)	0.309
	B	252	207	453	325	0.87 (0.69 - 1.11)	0.259
	C	9	450	37	741	0.40 (0.17 - 0.86)	0.012*
Dog is very interested in and adapts easily to new things and	A	1008	68	315	38	1.79 (1.15 - 2.76)	0.007**
	B	609	467	203	150	0.96 (0.75 - 1.24)	0.804
new places	C	34	1042	11	342	1.01 (0.50 - 2.24)	1.000
Dog behaves aggressively during visits to	A	93	12	1349	109	0.63 (0.33 - 1.30)	0.182
the veterinarian	B	59	46	819	639	1.00 (0.66 - 1.53)	1.000
	C	1	104	54	1404	0.25 (0.01 - 1.49)	0.175
Dog shows aggression when nervous or	A	325	31	1039	82	0.83 (0.53 - 1.32)	0.423
fearful	B	196	160	637	484	0.93 (0.73 - 1.19)	0.581
	C	8	348	39	1082	0.64 (0.26 - 1.40)	0.300
Dog takes a long time to lose	A	357	41	608	35	0.50 (0.30 - 0.82)	0.005**
interest in new things	B	201	197	386	257	0.68 (0.52 - 0.88)	0.003**
	C	9	389	23	620	0.62 (0.25 - 1.42)	0.271
Dog pants frequently, even at rest	A	189	16	1173	99	1.00 (0.57 - 1.85)	1.000
	B	116	89	716	556	1.01 (0.74 - 1.38)	1.000
	C	11	194	35	1237	2.00 (0.90 - 4.12)	0.052*

Dog is lethargic	A	132	9	1236	104	1.23 (0.61 - 2.84)	0.738
	B	89	52	731	609	1.43 (0.98 - 2.08)	0.061
	C	7	134	46	1294	1.47 (0.55 - 3.36)	0.339
Dog seems dull or depressed, not alert	A	32	3	1403	121	0.92 (0.28 - 4.76)	0.754
	B	16	19	853	671	0.66 (0.32 - 1.37)	0.234
	C	1	34	55	1469	0.79 (0.02 - 4.87)	1.000
Dog tends to be calm	A	890	76	334	22	0.77 (0.45 - 1.28)	0.344
	B	562	404	192	164	1.19 (0.92 - 1.53)	0.169
	C	32	934	19	337	0.61 (0.33 - 1.15)	0.107

Table S4. All ARG hits and the number of samples in which they were detected (A), higher-public-health-risk ARGs and the number of samples in which they were detected (B), and higher-public-health-risk ARGs derived from ESKAPE pathogens and the number of samples in which they were detected (C). MCR in the name is the abbreviation for mutation conferring resistance. *Escherichia coli* is abbreviated as E. coli, *Klebsiella pneumoniae* as K. pneumoniae, *Mycobacterium tuberculosis* as M. tuberculosis, *Pseudomonas aeruginosa* as P. aeruginosa, and *Staphylococcus aureus* as S. aureus.

ARG	A	B	C
23S rRNA (adenine(2058)-N(6))-methyltransferase Erm(A)	1		
AAC(3)-IId	1	1	
AAC(3)-IV	10	10	
AAC(6′)-29b	1		
AAC(6′)-Iaa	4		
AAC(6′)-Iak	4		
AAC(6′)-Ib7	2	2	
AAC(6′)-Ib9	2	2	
AAC(6′)-Ic	23	23	
AAC(6′)-Im	1	1	
AAC(6′)-Iy	4		
aad(6)	1	1	
aadA2	13	13	
aadA22	1	1	
aadA25	1		
aadA27	1		
aadA3	6	6	
aadA6	1	1	
aadA8b	1		
aadA9	2		
aadS	119		
abeM	1	1	1
acrB	13	13	4
acrD	11	11	2
AcrE	1		
AcrF	1		
AcrS	1		
ACT-2	1		
ACT-4	1		
ACT-56	1		
ADC-184	1		
adeF	1	1	1
adeG	1	1	1
adeH	1	1	1
adeI	1	1	
adeK	1	1	
almG	4		
ANT(2″)-Ia	19	19	3
ANT(6)-Ia	1	1	

ANT(6)-Ib	5	5	
APH(2″)-IIa	1	1	
APH(3″)-Ib	130	130	7
APH(3′)-Ia	54	54	5
APH(3′)-IIa	2	2	
APH(3′)-IIb	8	8	6
APH(3′)-IIIa	4	4	
APH(4)-Ia	5	5	
APH(6)-Ic	2	2	
APH(6)-Id	153	153	17
ArmR	29		
arnA	4	4	
ArnT	4		
AxyX	1	1	
bacA	12	12	3
baeR	12	12	4
baeS	1	1	
basR	7		
basS	7	7	5
bcr-1	6	6	4
BLMT	6		
BRO-2	1		
BRP(MBL)	29		
CARB-16	3	3	
CARB-50	2		
catA4	1		
catB11	1	1	
catII	13	13	
catIII	14	14	2
catS	8		
catV	28		
CfxA2	609	609	
CfxA3	44	44	
CfxA4	24	24	
CfxA5	7	7	
CfxA6	2		
chrB	37	37	
cmlA1	2	2	
cmlA5	8	8	1
cmlA9	2		
cmx	2	2	
cprR	9		
cprS	6		
cpxA	7	7	
CRP	75	75	3
CRP-1	1		
dfrA12	1	1	
dfrA14	12	12	4
dfrA15	2	2	
dfrA20	30		

dfrC	1	1	
dfrF	1	1	
DHA-12	1		
DHA-14	1		
EC-15	1		
E. coli EF-Tu MCR to Pulvomycin	1002		
emrA	1	1	
emrB	6	6	2
emrK	1	1	
emrR	16	16	4
emrY	1	1	
Enterobacter cloacae acrA	3		
eptA	1	1	
eptB	2		
EreA	1	1	
EreD	4		
Erm(41)	19		
ErmB	21	21	2
ErmF	202	202	
ErmG	4		
ErmX	7	7	
E. coli acrA	1		
E. coli AcrAB-TolC with AcrR MCR to ciprofloxacin, tetracycline, and ceftazidime	1		
E. coli AcrAB-TolC with MarR MCR to ciprofloxacin and tetracycline	4		
E. coli EF-Tu MCR to kirromycin	1		
E. coli emrE	1		
E. coli mdfA	1		
E. coli soxR with MCR	1		
E. coli soxS with MCR	9		
evgA	1	1	
evgS	1	1	
facT	4	4	
floR	1	1	
FosA	11		
FosA2	4	4	2
fosA5	1		
FosA6	2	2	1
FosK	2		
gadX	1	1	
E. coli GlpT with MCR to fosfomycin	13		
GOB-2	1		
H-NS	12	12	2
IND-6	1		
kdpE	1	1	
K. pneumoniae acrA	2		
K. pneumoniae KpnE	4		

K. pneumoniae KpnF	5		
K. pneumoniae KpnG	5		
K. pneumoniae KpnH	10		
K. pneumoniae OmpK37	3		
LEN-14	1	1	1
lnuA	1	1	1
lnuB	1	1	
lnuC	4	4	
LpsB	1		
LptD	4		
lsaE	1	1	
marA	22	22	5
MCR-9.1	1		
mdtA	1	1	
mdtB	9	9	1
mdtC	11	11	3
mdtE	1	1	
mdtF	1	1	
mdtG	1	1	
mdtH	1	1	
mdtM	1	1	
mdtN	1	1	
mdtO	1	1	
mdtP	1	1	
MecC-type methicillin resistance repressor MecI	2		
mecI	3	3	
Mef(En2)	68	68	
mel	18	18	
MexA	8	8	2
MexB	5	5	
MexC	7	7	5
MexD	4	4	2
MexE	5	5	3
MexF	5	5	3
MexG	12		
MexH	9		
MexI	3		
MexJ	8		
MexK	5		
MexL	9		
mexM	4	4	3
mexN	1	1	
mexP	7	7	5
mexQ	5	5	3
MexR	9		
MexS	6		
MexV	6		
MexW	6		

mexY	5	5	5
MexZ	8		
Morganella morganii gyrB conferring resistance to fluoroquinolones	2		
mphA	9	9	
mphE	4	4	2
msbA	14	14	3
msrE	4	4	2
mtrA	1	1	
MuxA	7		
MuxB	5		
MuxC	5		
M. tuberculosis intrinsic murA conferring resistance to fosfomycin	1		
nalC	10		
nalD	11		
nimA	9		
nimC	1		
nimF	109		
nimJ	1		
nimK	1		
OmpA	5		
OpmB	6	6	4
OpmD	3		
opmE	7	7	4
OpmH	9	9	5
OprJ	2	2	
OprM	6	6	2
OprN	5	5	3
oqxA	8	8	4
oqxB	2	2	1
OXA-114l	1		
OXA-119	1		
OXA-2	104	104	18
OXA-347	27		
OXA-494	3		
OXA-50	1	1	1
OXA-540	1		
OXA-789	1		
OXA-838	4		
OXA-846	1		
OXA-851	1		
OXA-903	2		
OXA-904	1		
OXY-1-2	1		
ParR	8		
ParS	7		
patB	1	1	
PDC-121	1		
PDC-23	1		

PDC-24	1		
PDC-3	2	2	1
PDC-39	1		
PDC-46	1		
PDC-5	1		
PDC-98	1		
pgpB	953		
PmpM	5	5	3
PmrF	1		
P. aeruginosa catB7	9		
P. aeruginosa CpxR	5		
P. aeruginosa emrE	9		
P. aeruginosa parE conferring resistance to fluoroquinolones	1		
qacE	2		
qacEdelta1	91		
qacG	3		
qacJ	1		
qacL	59		
qnrE1	1		
QnrS1	2	2	
RAHN-1	1		
ramA	9	9	2
rmtD2	1		
ROB-1	474		
ROB-10	127		
ROB-11	24		
ROB-12	7		
ROB-13	16		
ROB-2	22		
ROB-3	2		
ROB-5	1		
ROB-7	1		
rpsJ	7		
M. tuberculosis rpsL MCR to Streptomycin	12		
rsmA	15		
SAT-4	6	6	
SHV-1	1	1	
SHV-11	1	1	
SME-2	1		
smeR	1		
smeS	1	1	
P. aeruginosa soxR	11		
SRT-2	12		
SRT-3	12		
SST-1	1		
S. aureus LmrS	9		
sul1	54	54	15
sul2	97	97	6

sul3	2	2	
TEM-135	2	2	
TEM-178	2		
tet(32)	262		
tet(33)	2	2	
tet(37)	43		
tet(41)	21	21	
tet(42)	25	25	
tet(44)	1		
tet(A)	4	4	
tet(B)	2	2	
tet(C)	9	9	
tet(D)	1	1	
tet(H)	55		
tet(J)	1	1	
tet(M)	10		
tet(O)	138		
tet(Q)	426		
tet(W)	84		
tet(X)	77		
tet(X1)	4		
tet(X4)	61		
tet(X5)	49		
tet(X6)	6		
tet(Y)	12	12	
tetR	4		
TolC	1		
TriA	4		
TriB	4	4	2
TriC	4	4	2
Type B NfxB	10		
ugd	1	1	
E. coli UhpT with MCR to fosfomycin	18		
vanR gene in vanN cluster	1		
vanU gene in vanG cluster	7		
vanXY gene in vanC cluster	1		
vanXY gene in vanN cluster	1		
YajC	11		
YojI	1		

Table S5. Antibiotic groups and the number of samples in which ARGs were detected against them. Antibiotic compounds affected by multidrug resistance are displayed separately.

Drug Class	A	B	C
aminocoumarin	154	79	23
aminoglycoside	740	508	61
bicyclomycin	6	6	4
carbapenem	344	185	48
cephalosporin	1125	208	49
cephamycin	850	754	19
diaminopyrimidine	208	95	41
disinfecting agents and antiseptics	400	101	43
elfamycin	1007	4	
fluoroquinolone	365	163	56
fosfomycin	53	7	3
glycopeptide	45		
glycylcycline	237	54	16
lincosamide	301	277	3
macrolide	644	454	40
monobactam	162	60	16
nitrofuran	10	10	5
nitroimidazole	135	14	3
nucleoside	10	10	
oxazolidinone	9		
penam	1095	218	50
penem	148	55	12
peptide	1105	44	12
phenicol	375	164	54
pleuromutilin	1	1	
rifamycin	104	46	11
streptogramin A	253	230	2
streptogramin	277	253	4
streptogramin B	253	230	2
sulfonamide	215	172	25
tetracycline	1650	214	53

Table S6. The number of samples containing ARGs (ARG+) and lacking ARGs (ARG-) associated with questions regarding the behaviour of dogs involved in the survey. "Group 1" and "Group 2" represent answers "Never" and "Not never" (Always, Often, Sometimes, Rarely), respectively. In column "Approach", "A" indicates ARGs detected in any canine metagenomic samples, "B" is for higher-public-health-risk ARGs detected in any canine metagenomic samples, and "C" stands for higher-public-health-risk ARGs detected in ESKAPE pathogens. No stars is: *p*-value > 0.05 (not statistically significant), one star (*): *p*-value ≤ 0.05 (statistically significant at the 5% level).

Question	Approach	Number of samples				OR (95%CI)	p-value
		Never		Not never			
		AGR+	AGR-	AGR+	AGR-		
Dog howls	A	893	84	664	50	0.80 (0.54 - 1.17)	0.238
	B	546	432	402	312	0.98 (0.80 - 1.2)0	0.882
	C	35	943	24	690	1.07 (0.61 - 1.89)	0.894
Dog woo-woo barks	A	507	47	1055	84	0.86 (0.58 - 1.28)	0.439
	B	315	240	636	503	1.04 (0.84 - 1.28)	0.754
	C	15	540	45	1094	0.68 (0.35 - 1.25)	0.210
Dog whines when alone	A	588	41	971	93	1.37 (0.93 - 2.06)	0.113
	B	357	272	592	473	1.05 (0.86 - 1.29)	0.649
	C	23	606	35	1030	1.12 (0.62 - 1.97)	0.681
Dog whines to get attention, food, or toys	A	462	36	1047	94	1.15 (0.76 - 1.77)	0.551
	B	284	214	640	502	1.04 (0.84 - 1.30)	0.745
	C	19	479	40	1102	1.09 (0.59 - 1.95)	0.774
Dog eats grass	A	162	13	1385	121	1.09 (0.60 - 2.15)	0.883
	B	108	68	835	671	1.28 (0.92 - 1.79)	0.149
	C	9	167	50	1456	1.57 (0.67 - 3.30)	0.274
Dog buries toys / bones	A	1010	92	538	40	0.82 (0.54 - 1.22)	0.340
	B	621	482	320	258	1.04 (0.84 - 1.28)	0.718
	C	35	1068	23	555	0.79 (0.45 - 1.42)	0.401

Dog eats non-food items	A	586	44	912	84	1.23 (0.83 - 1.84)	0.300
	B	364	266	553	444	1.10 (0.89 - 1.35)	0.383
	C	22	608	36	961	0.97 (0.54 - 1.71)	1.000
Dog escapes from cages, kennels, or	A	852	71	694	61	1.05 (0.73 - 1.53)	0.785
enclosures	B	513	411	430	325	0.94 (0.77 - 1.15)	0.587
	C	37	887	23	732	1.33 (0.76 - 2.36)	0.355
Dog gets stuck behind objects and is unable	A	1048	93	498	39	0.88 (0.58 - 1.32)	0.561
to get around	B	626	515	317	221	0.85 (0.68 - 1.05)	0.126
	C	35	1106	25	513	0.65 (0.37 - 1.15)	0.121
Dog walks away or avoids being patted	A	705	50	841	80	1.34 (0.92 - 1.98)	0.120
	B	422	333	521	401	0.98 (0.80 - 1.19)	0.805
	C	36	719	24	898	1.87 (1.08 - 3.31)	0.024*
Dog shows barrier aggression	A	1037	82	507	50	1.25 (0.85 - 1.83)	0.249
	B	634	486	307	250	1.06 (0.86 - 1.31)	0.566
	C	43	1077	17	540	1.27 (0.70 - 2.39)	0.486
Dog lays in one place all day long	A	734	68	815	64	0.85 (0.58 - 1.23)	0.366
	B	434	368	513	367	0.84 (0.69 - 1.03)	0.085
	C	28	774	31	849	0.99 (0.57 - 1.72)	1.000
Dog damages doors, gates, or walls	A	1114	97	381	32	0.96 (0.62 - 1.48)	0.916
	B	687	525	228	185	1.06 (0.84 - 1.34)	0.606
	C	46	1166	13	400	1.21 (0.64 - 2.48)	0.648

Dog has difficulty finding food dropped on	A	751	62	794	68	1.04 (0.71 - 1.51)	0.856
the floor	B	455	358	486	376	0.98 (0.81 - 1.20)	0.883
	C	25	788	34	828	0.77 (0.44 - 1.35)	0.356
Dog licks his empty bowl after finishing	A	128	6	1410	124	1.88 (0.81 - 5.31)	0.177
the food	B	71	63	870	664	0.86 (0.59 - 1.25)	0.415
	C	5	129	54	1480	1.06 (0.33 - 2.70)	0.808
Dog licks tile / linoleum floors	A	558	42	983	89	1.20 (0.81 - 1.81)	0.393
	B	330	270	608	464	0.93 (0.76 - 1.15)	0.505
	C	19	581	40	1032	0.84 (0.46 - 1.51)	0.584
Dog turns in circles before pooping	A	383	34	1156	95	0.93 (0.61 - 1.44)	0.751
	B	241	176	696	555	1.09 (0.87 - 1.38)	0.459
	C	15	402	44	1207	1.02 (0.52 - 1.90)	1.000
Dog kicks or scratches the ground after	A	385	37	1153	91	0.82 (0.54 - 1.26)	0.342
defecating	B	238	184	698	546	1.01 (0.81 - 1.27)	0.955
	C	11	411	48	1196	0.67 (0.31 - 1.32)	0.286
Dog lifts his leg to urinate	A	598	46	935	84	1.17 (0.79 - 1.74)	0.454
	B	351	293	583	436	0.90 (0.73 - 1.10)	0.287
	C	21	623	36	983	0.92 (0.51 - 1.64)	0.890
Dog marks with feces	A	1034	91	502	39	0.88 (0.58 - 1.32)	0.560
	B	624	501	311	230	0.92 (0.74 - 1.14)	0.461
	C	42	1083	16	525	1.27 (0.69 - 2.45)	0.477

Dog eats his own feces	A	1310	108	230	21	1.11 (0.65 - 1.82)	0.700
	B	795	623	143	108	0.96 (0.73 - 1.27)	0.836
	C	50	1368	9	242	0.98 (0.47 - 2.30)	1.000
Dog shows submissive urination	A	1170	100	370	31	0.98 (0.62 - 1.51)	1.000
	B	726	544	214	187	1.17 (0.92 - 1.47)	0.185
	C	46	1224	13	388	1.12 (0.59 - 2.29)	0.877
Dog drinks water quickly	A	117	5	1371	122	2.08 (0.84 - 6.66)	0.117
	B	76	46	833	660	1.31 (0.88 - 1.96)	0.184
	C	4	118	54	1439	0.90 (0.23 - 2.51)	1.000
Dog rests frog style	A	512	37	1015	92	1.25 (0.83 - 1.92)	0.285
	B	309	240	621	486	1.01 (0.82 - 1.25)	0.958
	C	19	530	38	1069	1.01 (0.54 - 1.81)	1.000
Dog stares blankly at the walls or floor	A	909	83	610	47	0.84 (0.57 - 1.24)	0.402
	B	553	439	374	283	0.95 (0.78 - 1.17)	0.648
	C	34	958	24	633	0.94 (0.53 - 1.67)	0.892
Dog tilts his head	A	140	20	1384	110	0.56 (0.33 - 0.98)	0.029*
	B	92	68	838	656	1.06 (0.75 - 1.50)	0.801
	C	5	155	53	1441	0.88 (0.27 - 2.23)	1.000
Dog crosses his front paws	A	338	29	1185	100	0.98 (0.63 - 1.57)	0.913
	B	199	168	728	557	0.91 (0.71 - 1.15)	0.438
	C	10	357	48	1237	0.72 (0.32 - 1.46)	0.423
Dog shows handedness or side-preference	A	381	42	1140	87	0.69 (0.46 - 1.05)	0.074
	B	240	183	688	539	1.03 (0.82 - 1.29)	0.820
	C	12	411	45	1182	0.77 (0.37 - 1.49)	0.537

Dog places his paw on my or other people's feet	A	295	29	1177	98	0.85 (0.54 - 1.36)	0.490
	B	190	134	711	564	1.12 (0.87 - 1.45)	0.380
	C	10	314	47	1228	(0.83 0.37 - 1.69)	0.738

Table S7. The number of samples containing ARGs (ARG+) and lacking ARGs (ARG-) associated with questions regarding the behaviour of dogs involved in the survey. "Group 1" and "Group 2" represent answers "Agree" (Strongly agree, Agree) and "Disagree" (Strongly disagree, disagree), respectively. In column "Approach", "A" indicates ARGs detected in any canine metagenomic samples, "B" is for higher-public-health-risk ARGs detected in any canine metagenomic samples, and "C" stands for higher-public-health-risk ARGs detected in ESKAPE pathogens. No stars is: p-value > 0.05 (not statistically significant), one star (*): p-value $\leq$ 0.05 (statistically significant at the 5% level), two stars (**): p-value $\leq$ 0.01 (statistically significant at the 1% level) and three stars (***): p-value $\leq$ 0.001 (statistically significant at the 0.1% level).

Question	Approach	Number of samples				OR (95%CI)	p-value
		Agree		Disagree			
		AGR+	AGR-	AGR+	AGR-		
Dog enjoys life	A	1497	129	14	0	0.00 (0.00 - 3.55)	0.619
	B	905	722	10	4	0.50 (0.11 - 1.75)	0.288
	C	55	1572	1	13	0.46 (0.07 - 19.68)	0.386
Dog wants to play	A	1295	117	93	4	0.48 (0.12 - 1.30)	0.176
	B	779	634	66	31	0.58 (0.36 - 0.91)	0.015*
	C	48	1365	7	90	0.45 (0.20 - 1.22)	0.082
Dog becomes aggressive when excited	A	262	22	1075	93	1.03 (0.63 - 1.76)	1.000
	B	144	140	681	488	0.74 (0.56 - 0.96)	0.023*
	C	7	277	43	1126	0.66 (0.25 - 1.51)	0.369
Dog enjoys playing with toys	A	1228	110	203	11	0.61 (0.29 - 1.15)	0.132
	B	738	601	131	83	0.78 (0.57 - 1.06)	0.103
	C	47	1292	10	204	0.74 (0.36 - 1.67)	0.431
Dog gets bored in play quickly	A	389	27	823	87	1.52 (0.96 - 2.48)	0.072
	B	242	174	491	420	1.19 (0.93 - 1.52)	0.153
	C	13	403	37	874	0.76 (0.37 - 1.49)	0.442
Dog seeks constant activity	A	394	39	880	69	0.79 (0.52 - 1.23)	0.280
	B	208	226	578	371	0.59 (0.47 - 0.75)	0.000***
	C	14	420	37	912	0.82 (0.41 - 1.58)	0.645

Excitement can lead dog to fixed repetitive behavior	A	318	22	1002	92	1.33 (0.81 - 2.26)	0.301
	B	188	153	631	463	0.90 (0.70 - 1.16)	0.416
	C	16	325	38	1056	1.37 (0.70 - 2.55)	0.328
Dog calms down very quickly after being	A	1039	89	268	23	1.00 (0.59 - 1.64)	1.000
excited	B	640	489	165	126	1.00 (0.76 - 1.31)	1.000
	C	40	1089	9	282	1.15 (0.54 - 2.73)	0.857
Dog may become aggressive if he is	A	197	22	1208	104	0.77 (0.47 - 1.32)	0.289
frustrated with something	B	114	105	748	565	0.82 (0.61 - 1.10)	0.186
	C	6	213	43	1270	0.83 (0.29 - 2.00)	0.836
Dog is not very patient	A	422	39	817	74	0.98 (0.64 - 1.51)	0.918
	B	263	198	505	387	1.02 (0.81 - 1.29)	0.908
	C	18	443	28	864	1.25 (0.65 - 2.38)	0.527
Dog is relaxed when greeting people	A	491	32	882	92	1.60 (1.04 - 2.51)	0.030*
	B	315	208	519	456	1.33 (1.07 - 1.66)	0.010*
	C	20	503	34	941	1.10 (0.59 - 1.99)	0.772
Dog is shy	A	402	45	980	81	0.74 (0.50 - 1.11)	0.127
	B	243	204	601	461	0.91 (0.73 - 1.15)	0.427
	C	14	433	38	1024	0.87 (0.43 - 1.67)	0.758
Dog is boisterous	A	684	63	578	47	0.88 (0.58 - 1.33)	0.551
	B	392	356	365	260	0.78 (0.63 - 0.98)	0.029*
	C	31	717	16	609	1.65 (0.86 - 3.25)	0.136

Dog does not think before he acts	A	481	36	744	57	1.02 (0.65 - 1.63)	1.000
	B	281	236	465	336	0.86 (0.68 - 1.08)	0.191
	C	20	497	33	768	0.94 (0.50 - 1.70)	0.886
Dog appears to be sorry after he has done something wrong	A	956	82	268	27	1.17 (0.72 - 1.88)	0.472
	B	563	476	173	122	0.83 (0.64 - 1.09)	0.185
	C	34	1005	14	281	0.68 (0.35 - 1.39)	0.220
Dog aggressively guards coveted items (e.g., stolen item, treats, food bowl)	A	295	26	1098	95	0.98 (0.62 - 1.61)	0.908
	B	174	147	677	517	0.90 (0.70 - 1.17)	0.447
	C	13	308	42	1152	1.16 (0.56 - 2.23)	0.617
Dog is able to focus on a task in a distracting situation (e.g., loud or busy places, around other dogs)	A	645	56	600	50	0.96 (0.63 - 1.46)	0.919
	B	403	298	348	303	1.18 (0.94 - 1.47)	0.140
	C	27	674	22	629	1.15 (0.62 - 2.13)	0.665
Dog is playful with other dogs	A	946	85	314	22	0.78 (0.46 - 1.28)	0.351
	B	564	468	204	132	0.78 (0.60 - 1.01)	0.058
	C	37	995	14	322	0.86 (0.44 - 1.74)	0.620
When off leash, dog comes immediately when called	A	653	56	549	44	0.93 (0.60 - 1.44)	0.755
	B	383	326	342	251	0.86 (0.69 - 1.08)	0.198
	C	25	684	28	565	0.74 (0.41 - 1.33)	0.325

Dog likes to chase squirrels, birds, or other small animals	A	1188	105	227	20	1.00 (0.57 - 1.66)	1.000
	B	725	569	143	104	0.93 (0.70 - 1.23)	0.624
	C	37	1257	16	231	0.43 (0.23 - 0.83)	0.007**
Dog moves normally	A	1388	118	114	11	1.13 (0.54 - 2.19)	0.729
	B	833	674	76	49	0.80 (0.54 - 1.17)	0.261
	C	52	1455	4	121	1.08 (0.39 - 4.19)	1.000
Dog is as active as he has been	A	1206	115	248	12	0.51 (0.25 - 0.94)	0.025*
	B	716	606	174	86	0.58 (0.44 - 0.78)	0.000***
	C	46	1276	10	250	0.90 (0.44 - 2.03)	0.716
Dog shows extreme physical signs when	A	766	62	422	42	1.23 (0.80 - 1.88)	0.33
excited	B	456	373	280	184	0.80 (0.63 - 1.02)	0.070
	C	27	802	21	443	0.71 (0.38 - 1.34)	0.283
Dog ignores commands	A	333	21	846	83	1.56 (0.94 - 2.69)	0.086
	B	196	158	512	418	1.01 (0.79 - 1.31)	0.950
	C	18	336	30	900	1.61 (0.83 - 3.02)	0.138
Dog is quick to sneak out through open	A	375	34	922	79	0.95 (0.61 - 1.48)	0.829
doors, gates	B	225	184	574	427	0.91 (0.72 - 1.16)	0.442
	C	12	397	41	960	0.71 (0.34 - 1.39)	0.356
Dog sleeps more, is less awake	A	509	37	675	58	1.18 (0.76 - 1.87)	0.453
	B	335	211	390	344	1.40 (1.11 - 1.77)	0.004**
	C	19	527	35	699	0.72 (0.38 - 1.31)	0.325

Dog is slow to respond to corrections	A	193	15	1085	99	1.17 (0.66 - 2.22)	0.681
	B	116	92	667	518	0.98 (0.72 - 1.33)	0.940
	C	9	199	45	1140	1.15 (0.48 - 2.42)	0.697
Dog doesn't like to be approached or	A	319	30	1012	77	0.81 (0.51 - 1.30)	0.349
hugged	B	195	154	615	475	0.98 (0.76 - 1.26)	0.901
	C	12	337	42	1048	0.89 (0.42 - 1.74)	0.872
Dog is friendly towards unfamiliar people	A	942	71	390	39	1.33 (0.86 - 2.03)	0.193
	B	577	437	245	184	0.99 (0.78 - 1.25)	0.954
	C	36	978	13	416	1.18 (0.60 - 2.45)	0.751
Dog responds to my presence	A	1532	130	4	2	5.88 (0.53 - 41.46)	0.075
	B	931	732	2	4	2.54 (0.36 - 28.18)	0.415
	C	58	1605	0	6	Inf (0.04 - Inf)	1.000
Dog behaves aggressively in response to	A	286	34	1071	82	0.64 (0.42 - 1.01)	0.046*
perceived threats from people (e.g., being	B	175	145	656	498	0.92 (0.71 - 1.19)	0.524
cornered, having collar reached for)	C	9	311	44	1110	0.73 (0.31 - 1.54)	0.498
Dog is a people person	A	1115	86	237	24	1.31 (0.78 - 2.14)	0.247
	B	684	518	155	106	0.90 (0.68 - 1.20)	0.490
	C	46	1156	8	253	1.26 (0.58 - 3.13)	0.717

Dog behaves fearfully towards unfamiliar people	A	413	51	942	68	0.58 (0.39 - 0.87)	0.007**
	B	249	216	593	417	0.81 (0.65 - 1.02)	0.070
	C	15	450	38	972	0.85 (0.43 - 1.61)	0.655
Dog seems to get excited for no reason	A	291	26	1031	86	0.93 (0.58 - 1.54)	0.812
	B	174	143	651	467	0.87 (0.67 - 1.13)	0.303
	C	11	306	42	1076	0.92 (0.42 - 1.85)	1.000
Dog behaves aggressively towards unfamiliar people	A	192	27	1211	91	0.53 (0.33 - 0.88)	0.009**
	B	126	93	748	555	1.01 (0.75 - 1.36)	1.000
	C	4	215	51	1252	0.46 (0.12 - 1.26)	0.168
Dog seeks companionship from people	A	1295	107	109	10	1.11 (0.50 - 2.20)	0.721
	B	784	619	69	50	0.92 (0.61 - 1.36)	0.701
	C	51	1352	4	115	1.08 (0.39 - 4.20)	1.000
Dog must greet everyone who comes to the door	A	1245	102	168	18	1.31 (0.73 - 2.24)	0.309
	B	765	583	106	80	0.99 (0.72 - 1.36)	1.000
	C	48	1300	7	179	0.94 (0.42 - 2.51)	0.834
Dog chases bicycles, joggers, and skateboarders	A	311	22	1014	80	1.12 (0.68 - 1.91)	0.717
	B	192	141	612	482	1.07 (0.83 - 1.39)	0.614
	C	10	323	40	1054	0.82 (0.36 - 1.68)	0.733

Dog works at tasks until entirely finished	A	1121	103	248	15	0.66 (0.35 - 1.16)	0.166
	B	679	545	150	113	0.94 (0.71 - 1.24)	0.682
	C	43	1181	10	253	0.92 (0.45 - 2.08)	0.854
Dog leaves food or objects alone when told	A	1068	95	236	20	0.95 (0.55 - 1.59)	1.000
to do so	B	645	518	146	110	0.94 (0.71 - 1.24)	0.677
	C	38	1125	12	244	0.69 (0.35 - 1.47)	0.263
Dog retrieves objects (e.g., balls, toys,	A	961	86	409	29	0.79 (0.49 - 1.24)	0.338
sticks)	B	564	483	275	163	0.69 (0.55 - 0.88)	0.002**
	C	38	1009	15	423	1.06 (0.56 - 2.10)	1.000
Dog appears to have a lot of control over	A	992	83	236	19	0.96 (0.54 - 1.64)	1.000
how he responds	B	611	464	144	111	1.02 (0.76 - 1.35)	0.944
	C	42	1033	5	250	2.03 (0.79 - 6.65)	0.184
Dog reacts very quickly	A	1217	102	107	10	1.11 (0.50 - 2.22)	0.719
	B	720	599	74	43	0.70 (0.46 - 1.05)	0.081
	C	42	1277	5	112	0.74 (0.28 - 2.43)	0.583
Dog is easy to train	A	1068	93	204	14	0.79 (0.41 - 1.42)	0.491
	B	643	518	132	86	0.81 (0.59 - 1.10)	0.180
	C	42	1119	9	209	0.87 (0.41 - 2.07)	0.696
Dog can be very persistent	A	1257	110	103	8	0.89 (0.36 - 1.88)	0.857
	B	760	607	70	41	0.73 (0.48 - 1.11)	0.136
	C	50	1317	4	107	1.02 (0.36 - 3.95)	1.000

Dog points	A	365	26	917	82	1.26 (0.78 - 2.07)	0.373
	B	225	166	565	434	1.04 (0.82 - 1.33)	0.763
	C	12	379	39	960	0.78 (0.37 - 1.54)	0.528
Dog avoids getting wet	A	609	46	690	63	1.21 (0.80 - 1.84)	0.369
	B	385	270	408	345	1.21 (0.97 - 1.50)	0.085
	C	24	631	24	729	1.16 (0.62 - 2.15)	0.660
Dog behaves aggressively toward other	A	322	33	917	78	0.83 (0.53 - 1.31)	0.431
dogs	B	198	157	557	438	0.99 (0.77 - 1.28)	0.950
	C	6	349	46	949	0.35 (0.12 - 0.84)	0.015*
Dog willingly shares his toys with other	A	681	51	425	41	1.29 (0.82 - 2.02)	0.266
dogs	B	409	323	276	190	0.87 (0.68 - 1.11)	0.256
	C	27	705	19	447	0.90 (0.48 - 1.74)	0.759
Dog is dominant over other dogs	A	428	36	696	62	1.06 (0.68 - 1.67)	0.829
	B	265	199	421	337	1.07 (0.84 - 1.36)	0.594
	C	16	448	29	729	0.90 (0.45 - 1.73)	0.876
Dog avoids other dogs	A	200	15	1099	96	1.16 (0.66 - 2.21)	0.681
	B	130	85	649	546	1.29 (0.95 - 1.75)	0.101
	C	9	206	47	1148	1.07 (0.45 - 2.25)	0.850
Dog is friendly towards other dogs	A	988	82	217	21	1.17 (0.67 - 1.95)	0.594
	B	608	462	130	108	1.09 (0.82 - 1.46)	0.563
	C	41	1029	6	232	1.54 (0.64 - 4.49)	0.441

Dog knows he is a dog	A	989	76	152	19	1.63 (0.90 - 2.81)	0.087
	B	596	469	95	76	1.02 (0.72 - 1.42)	0.934
	C	39	1026	9	162	0.68 (0.32 - 1.64)	0.290
Dog behaves fearfully towards other dogs	A	208	26	1087	87	0.64 (0.40 - 1.06)	0.065
	B	126	108	665	509	0.89 (0.67 - 1.20)	0.471
	C	7	227	44	1130	0.79 (0.30 - 1.80)	0.703
Dog behaves aggressively towards cats	A	421	38	726	52	0.79 (0.50 - 1.26)	0.309
	B	252	207	453	325	0.87 (0.69 - 1.11)	0.259
	C	9	450	37	741	0.40 (0.17 - 0.86)	0.012*
Dog is assertive or pushy with other dogs	A	519	38	709	66	1.27 (0.83 - 1.98)	0.301
	B	306	251	450	325	0.88 (0.70 - 1.10)	0.263
	C	18	539	29	746	0.86 (0.44 - 1.62)	0.654
Dog sometimes fails to recognize familiar people or pets	A	111	16	1275	104	0.57 (0.32 - 1.06)	0.057
	B	69	58	781	598	0.91 (0.62 - 1.34)	0.641
	C	5	122	51	1328	1.07 (0.33 - 2.73)	0.807
Dog is very interested in and adapts easily to new things and new places	A	1008	68	315	38	1.79 (1.15 - 2.76)	0.007**
	B	609	467	203	150	0.96 (0.75 - 1.24)	0.804
	C	34	1042	11	342	1.01 (0.50 - 2.24)	1.000

Dog exhibits fearful behaviors when he is restrained	A	315	30	974	79	0.85 (0.54 - 1.37)	0.488
	B	190	155	603	450	0.91 (0.71 - 1.18)	0.491
	C	11	334	41	1012	0.81 (0.37 - 1.63)	0.625
Dog behaves fearfully when groomed (e.g., nails trimmed, brushed, bathed, ears cleaned)	A	531	47	755	54	0.81 (0.53 - 1.24)	0.346
	B	325	253	465	344	0.95 (0.76 - 1.19)	0.660
	C	18	560	31	778	0.81 (0.42 - 1.51)	0.556
Dog is not keen to go into new situations	A	392	43	896	68	0.69 (0.46 - 1.06)	0.087
	B	239	196	550	414	0.92 (0.73 - 1.16)	0.485
	C	13	422	36	928	0.79 (0.38 - 1.55)	0.533
Dog behaves aggressively during visits to the veterinarian	A	93	12	1349	109	0.63 (0.33 - 1.30)	0.182
	B	59	46	819	639	1.00 (0.66 - 1.53)	1.000
	C	1	104	54	1404	0.25 (0.01 - 1.49)	0.175
Dog is highly sensitive to noise	A	626	55	667	54	0.92 (0.61 - 1.39)	0.691
	B	380	301	413	308	0.94 (0.76 - 1.17)	0.590
	C	20	661	30	691	0.70 (0.37 - 1.28)	0.250
Dog shows aggression when nervous or fearful	A	325	31	1039	82	0.83 (0.53 - 1.32)	0.423
	B	196	160	637	484	0.93 (0.73 - 1.19)	0.581
	C	8	348	39	1082	0.64 (0.26 - 1.40)	0.300

Dog behaves fearfully during visits to the	A	506	41	853	71	1.03 (0.68 - 1.57)	0.919
veterinarian	B	317	230	518	406	1.08 (0.87 - 1.35)	0.514
	C	14	533	38	886	0.61 (0.30 - 1.17)	0.144
Dog takes a long time to lose interest in	A	357	41	608	35	0.50 (0.30 - 0.82)	0.005**
new things	B	201	197	386	257	0.68 (0.52 - 0.88)	0.003**
	C	9	389	23	620	0.62 (0.25 - 1.42)	0.271
Dog is afraid of storms	A	457	34	885	68	1.03 (0.66 - 1.63)	0.914
	B	276	215	541	412	0.98 (0.78 - 1.23)	0.867
	C	16	475	38	915	0.81 (0.42 - 1.51)	0.560
Dog behaves submissively (e.g., rolls over,	A	221	13	1136	99	1.48 (0.81 - 2.93)	0.227
avoids eye contact, licks his lips) when	B	134	100	696	539	1.04 (0.78 - 1.39)	0.829
greeting other dogs	C	7	227	46	1189	0.80 (0.30 - 1.81)	0.704
Dog paces up and down, walks in circles	A	172	14	1230	100	1.00 (0.55 - 1.94)	1.000
and/or wanders with no direction or	B	107	79	748	582	1.05 (0.76 - 1.46)	0.753
purpose	C	5	181	49	1281	0.72 (0.22 - 1.84)	0.672
Dog pants frequently, even at rest	A	189	16	1173	99	1.00 (0.57 - 1.85)	1.000
	B	116	89	716	556	1.01 (0.74 - 1.38)	1.000
	C	11	194	35	1237	2.00 (0.90 - 4.12)	0.052*

Dog shakes or trembles occasionally	A	499	46	898	73	0.88 (0.59 - 1.33)	0.551
	B	317	228	523	448	1.19 (0.96 - 1.48)	0.107
	C	18	527	32	939	1.00 (0.52 - 1.86)	1.000
Dog is lethargic	A	132	9	1236	104	1.23 (0.61 - 2.84)	0.738
	B	89	52	731	609	1.43 (0.98 - 2.08)	0.061
	C	7	134	46	1294	1.47 (0.55 - 3.36)	0.339
Dog is confident	A	1038	81	219	21	1.23 (0.71 - 2.06)	0.419
	B	642	477	132	108	1.10 (0.82 - 1.47)	0.518
	C	44	1075	6	234	1.60 (0.67 - 4.64)	0.348
Dog seems dull or depressed, not alert	A	32	3	1403	121	0.92 (0.28 - 4.76)	0.754
	B	16	19	853	671	0.66 (0.32 - 1.37)	0.234
	C	1	34	55	1469	0.79 (0.02 - 4.87)	1.000
Dog is anxious	A	502	43	736	63	1.00 (0.66 - 1.53)	1.000
	B	308	237	445	354	1.03 (0.83 - 1.30)	0.780
	C	12	533	32	767	0.54 (0.25 - 1.09)	0.085
Dog is considered to be very impulsive	A	421	30	789	70	1.24 (0.79 - 2.01)	0.381
	B	242	209	501	358	0.83 (0.65 - 1.05)	0.113
	C	14	437	30	829	0.89 (0.43 - 1.74)	0.750
Dog is curious	A	1361	113	46	4	1.05 (0.27 - 2.95)	0.790
	B	822	652	31	19	0.77 (0.41 - 1.43)	0.469
	C	54	1420	2	48	0.91 (0.23 - 7.95)	0.706
Dog is affectionate	A	1392	121	33	2	0.70 (0.08 - 2.78)	1.000
	B	846	667	23	12	0.66 (0.30 - 1.40)	0.302
	C	56	1457	1	34	1.31 (0.21 - 54.05)	1.000

Dog tends to be calm	A	890	76	334	22	0.77 (0.45 - 1.28)	0.344
	B	562	404	192	164	1.19 (0.92 - 1.53)	0.169
	C	32	934	19	337	0.61 (0.33 - 1.15)	0.107
Dog is aloof	A	251	24	1045	89	0.89 (0.55 - 1.49)	0.621
	B	162	113	629	505	1.15 (0.87 - 1.52)	0.310
	C	6	269	45	1089	0.54 (0.19 - 1.29)	0.206
Dog has more good days than bad days	A	1377	117	34	4	1.38 (0.35 - 3.98)	0.536
	B	840	654	26	12	0.59 (0.27 - 1.23)	0.140
	C	53	1441	3	35	0.43 (0.13 - 2.25)	0.159

Table S8. The number of samples containing ARGs (ARG+) and lacking ARGs (ARG-) associated with questions regarding the behaviour of dogs involved in the survey. "Group 1" and "Group 2" represent answers "Agree" (Strongly agree, Agree), "Neither agree, nor disagree", and "Disagree" (Strongly disagree, disagree), respectively. In column "Approach", "A" indicates ARGs detected in any canine metagenomic samples, "B" is for higher-public-health-risk ARGs detected in any canine metagenomic samples, and "C" stands for higher-public-health-risk ARGs detected in ESKAPE pathogens. No stars is: *p*-value > 0.05 (not statistically significant), one star (*): *p*-value ≤ 0.05 (statistically significant at the 5% level), two stars (**): *p*-value ≤ 0.01 (statistically significant at the 1% level) and three stars (***): *p*-value ≤ 0.001 (statistically significant at the 0.1% level).

Question	Approach	Number of samples						p-value
		Agree		Neither		Disagree		
		ARG+	ARG-	ARG+	ARG-	ARG+	ARG-	
Dog enjoys life	A	1497	129	60	4	14	0	0.741
	B	905	722	43	21	10	4	0.095
	C	55	1572	3	61	1	13	0.332
Dog wants to play	A	1295	117	177	10	93	4	0.167
	B	779	634	109	78	66	31	0.037
	C	48	1365	4	183	7	90	0.097
Dog becomes aggressive when excited	A	262	22	226	16	1075	93	0.815
	B	144	140	125	117	681	488	0.024*
	C	7	277	8	234	43	1126	0.644
Dog enjoys playing with toys	A	1228	110	140	13	203	11	0.283
	B	738	601	88	65	131	83	0.233
	C	47	1292	2	151	10	204	0.216
Dog gets bored in play quickly	A	389	27	349	16	823	87	0.004**
	B	242	174	218	147	491	420	0.108
	C	13	403	9	356	37	874	0.348
Dog seeks constant activity	A	394	39	288	26	880	69	0.499
	B	208	226	166	148	578	371	0.000***
	C	14	420	7	307	37	912	0.390
Excitement can lead DOG to fixed repetitive behavior	A	318	22	242	19	1002	92	0.503
	B	188	153	135	126	631	463	0.198
	C	16	325	5	256	38	1056	0.181

Dog calms down very quickly after being excited	A	1039	89	253	20	268	23	0.972
	B	640	489	145	128	165	126	0.557
	C	40	1089	10	263	9	282	0.922
Dog may become aggressive if he is frustrated with something	A	197	22	156	8	1208	104	0.174
	B	114	105	91	73	748	565	0.384
	C	6	213	10	154	43	1270	0.164
Dog is not very patient	A	422	39	275	18	817	74	0.456
	B	263	198	162	131	505	387	0.892
	C	18	443	12	281	28	864	0.618
Dog is relaxed when greeting people	A	491	32	193	10	882	92	0.019*
	B	315	208	120	83	519	456	0.022*
	C	20	503	6	197	34	941	0.877
Dog is shy	A	402	45	171	8	980	81	0.053*
	B	243	204	102	77	601	461	0.702
	C	14	433	8	171	38	1024	0.677
Dog is boisterous	A	684	63	298	24	578	47	0.786
	B	392	356	192	130	365	260	0.029*
	C	31	717	13	309	16	609	0.233
Dog does not think before he acts	A	481	36	338	41	744	57	0.066
	B	281	236	205	175	465	336	0.274
	C	20	497	7	373	33	768	0.112
Dog appears to be sorry after he has done something wrong	A	956	82	333	25	268	27	0.594
	B	563	476	213	145	173	122	0.137
	C	34	1005	10	348	14	281	0.363

Dog aggressively guards coveted items (e.g., stolen item, treats, food bowl)	A	295	26	165	13	1098	95	0.968
	B	174	147	99	79	677	517	0.714
	C	13	308	5	173	42	1152	0.793
Dog is able to focus on a task in a distracting situation (e.g., loud or busy places, around other dogs)	A	645	56	313	27	600	50	0.979
	B	403	298	198	142	348	303	0.221
	C	27	674	10	330	22	629	0.769
Dog is playful with other dogs	A	946	85	289	25	314	22	0.633
	B	564	468	174	140	204	132	0.146
	C	37	995	7	307	14	322	0.370
When off leash, dog comes immediately when called	A	653	56	345	30	549	44	0.930
	B	383	326	221	155	342	251	0.235
	C	25	684	6	370	28	565	0.030
Dog likes to chase squirrels, birds, or other small animals	A	1188	105	133	9	227	20	0.821
	B	725	569	73	69	143	104	0.458
	C	37	1257	5	137	16	231	0.024
Dog moves normally	A	1388	118	54	5	114	11	0.851
	B	833	674	38	21	76	49	0.208
	C	52	1455	3	56	4	121	0.698

Dog is as active as he has been	A	1206	115	89	5	248	12	0.052*
	B	716	606	51	43	174	86	0.001**
	C	46	1276	3	91	10	250	0.935
Dog shows extreme physical signs when excited	A	766	62	358	29	422	42	0.566
	B	456	373	205	182	280	184	0.069
	C	27	802	11	376	21	443	0.372
Dog ignores commands	A	333	21	377	28	846	83	0.157
	B	196	158	240	165	512	418	0.343
	C	18	336	12	393	30	900	0.219
Dog is quick to sneak out through open doors, gates	A	375	34	249	19	922	79	0.857
	B	225	184	144	125	574	427	0.459
	C	12	397	7	262	41	960	0.430
Dog sleeps more, is less awake	A	509	37	361	34	675	58	0.551
	B	335	211	217	178	390	344	0.011*
	C	19	527	6	389	35	699	0.015*
Dog is slow to respond to corrections	A	193	15	269	16	1085	99	0.295
	B	116	92	163	122	667	518	0.948
	C	9	199	6	279	45	1140	0.294
Dog doesn't like to be approached or hugged	A	319	30	224	24	1012	77	0.290
	B	195	154	137	111	615	475	0.942
	C	12	337	5	243	42	1048	0.385
Dog is friendly towards unfamiliar people	A	942	71	223	22	390	39	0.295
	B	577	437	126	119	245	184	0.270
	C	36	978	10	235	13	416	0.756

Dog responds to my presence	A	1532	130	18	0	4	2	0.054*
	B	931	732	14	4	2	4	0.097
	C	58	1605	1	17	0	6	0.577
Dog behaves aggressively in response to perceived threats from people	A	286	34	195	14	1071	82	0.109
	B	175	145	114	95	656	498	0.692
	C	9	311	5	204	44	1110	0.547
Dog is a people person	A	1115	86	196	22	237	24	0.205
	B	684	518	107	111	155	106	0.056
	C	46	1156	4	214	8	253	0.371
Dog behaves fearfully towards unfamiliar people	A	413	51	196	13	942	68	0.015*
	B	249	216	104	105	593	417	0.025*
	C	15	450	5	204	38	972	0.624
Dog seems to get excited for no reason	A	291	26	228	18	1031	86	0.925
	B	174	143	119	127	651	467	0.017*
	C	11	306	6	240	42	1076	0.690
Dog behaves aggressively towards unfamiliar people	A	192	27	146	14	1211	91	0.026*
	B	126	93	72	88	748	555	0.011*
	C	4	215	4	156	51	1252	0.291
DOG seeks companionship from people	A	1295	107	150	14	109	10	0.826
	B	784	619	96	68	69	50	0.769
	C	51	1352	4	160	4	115	0.832

Dog must greet everyone who comes to the door	A	1245	102	82	9	168	18	0.406
	B	765	583	45	46	106	80	0.394
	C	48	1300	2	89	7	179	0.854
Dog chases bicycles, joggers, and skateboarders	A	311	22	225	29	1014	80	0.070
	B	192	141	136	118	612	482	0.612
	C	10	323	9	245	40	1054	0.906
Dog works at tasks (e.g., getting treats out of a Kong, shredding toys) until entirely finished	A	1121	103	171	10	248	15	0.192
	B	679	545	106	75	150	113	0.706
	C	43	1181	5	176	10	253	0.860
Dog leaves food or objects alone when told to do so	A	1068	95	242	16	236	20	0.610
	B	645	518	148	110	146	110	0.803
	C	38	1125	9	249	12	244	0.487
Dog retrieves objects (e.g., balls, toys, sticks)	A	961	86	177	15	409	29	0.610
	B	564	483	100	92	275	163	0.0033*
	C	38	1009	5	187	15	423	0.866
Dog appears to have a lot of control over how he responds	A	992	83	315	29	236	19	0.879
	B	611	464	180	164	144	111	0.332
	C	42	1033	12	332	5	250	0.346
Dog reacts very quickly	A	1217	102	223	19	107	10	0.889
	B	720	599	145	97	74	43	0.080
	C	42	1277	12	230	5	112	0.328

Dog is easy to train	A	1068	93	271	24	204	14	0.732
	B	643	518	162	133	132	86	0.343
	C	42	1119	8	287	9	209	0.686
Dog can be very persistent	A	1257	110	186	13	103	8	0.769
	B	760	607	108	91	70	41	0.278
	C	50	1317	4	195	4	107	0.525
Dog points	A	365	26	258	23	917	82	0.630
	B	225	166	144	137	565	434	0.215
	C	12	379	8	273	39	960	0.659
Dog avoids getting wet	A	609	46	189	19	690	63	0.488
	B	385	270	114	94	408	345	0.204
	C	24	631	8	200	24	729	0.798
Dog behaves aggres-sively toward other dogs	A	322	33	297	18	917	78	0.227
	B	198	157	181	134	557	438	0.887
	C	6	349	7	308	46	949	0.014
Dog willingly shares his toys with other	A	681	51	418	36	425	41	0.502
dogs	B	409	323	245	209	276	190	0.260
	C	27	705	12	442	19	447	0.459
Dog is dominant over other dogs	A	428	36	405	31	696	62	0.816
	B	265	199	248	188	421	337	0.842
	C	16	448	14	422	29	729	0.873
Dog avoids other dogs	A	200	15	237	18	1099	96	0.855
	B	130	85	156	99	649	546	0.053*
	C	9	206	3	252	47	1148	0.063
Dog is friendly towards other dogs	A	988	82	325	24	217	21	0.673
	B	608	462	196	153	130	108	0.823
	C	41	1029	12	337	6	232	0.685

Dog knows he is a dog	A	989	76	389	33	152	19	0.199
	B	596	469	241	181	95	76	0.909
	C	39	1026	10	412	9	162	0.185
Dog behaves fearfully towards other dogs	A	208	26	237	14	1087	87	0.069
	B	126	108	143	108	665	509	0.713
	C	7	227	8	243	44	1130	0.873
Dog behaves aggressively towards cats	A	421	38	385	37	726	52	0.342
	B	252	207	230	192	453	325	0.351
	C	9	450	13	409	37	741	0.032
Dog is assertive or pushy with other dogs (e.g.,if in a home with other dogs, when greeting)	A	519	38	302	24	709	66	0.526
	B	306	251	177	149	450	325	0.380
	C	18	539	11	315	29	746	0.894
Dog sometimes fails to recognize familiar people or pets	A	111	16	91	4	1275	104	0.062
	B	69	58	56	39	781	598	0.791
	C	5	122	2	93	51	1328	0.766
Dog is very interested in and adapts easily to new things and new places	A	1008	68	209	24	315	38	0.008**
	B	609	467	123	110	203	150	0.494
	C	34	1042	14	219	11	342	0.110
Dog exhibits fearful behaviors when he is restrained	A	315	30	236	18	974	79	0.712
	B	190	155	137	117	603	450	0.554
	C	11	334	7	247	41	1012	0.678

Dog behaves fearfully when groomed (e.g., nails trimmed, brushed, bathed, ears cleaned)	A	531	47	244	28	755	54	0.145
	B	325	253	141	131	465	344	0.270
	C	18	560	9	263	31	778	0.776
Dog is not keen to go into new situations	A	392	43	236	16	896	68	0.138
	B	239	196	144	108	550	414	0.745
	C	13	422	10	242	36	928	0.735
Dog behaves aggressively during visits to the veterinarian	A	93	12	77	9	1349	109	0.190
	B	59	46	47	39	819	639	0.958
	C	1	104	2	84	54	1404	0.319
Dog is highly sensitive to noise	A	626	55	234	21	667	54	0.880
	B	380	301	140	115	413	308	0.760
	C	20	661	9	246	30	691	0.478
Dog shows aggression when nervous or fearful	A	325	31	159	17	1039	82	0.413
	B	196	160	96	80	637	484	0.747
	C	8	348	10	166	39	1082	0.136
Dog behaves fearfully during visits to the veterinarian	A	506	41	168	17	853	71	0.716
	B	317	230	95	90	518	406	0.296
	C	14	533	6	179	38	886	0.287
Dog takes a long time to lose interest in new things	A	357	41	564	54	608	35	0.009 **
	B	201	197	346	272	386	257	0.011 *
	C	9	389	26	592	23	620	0.253

Dog is afraid of storms	A	457	34	139	25	885	68	0.003 **
	B	276	215	89	75	541	412	0.833
	C	16	475	4	160	38	915	0.631
Dog behaves submissively when greeting other dogs	A	221	13	173	18	1136	99	0.287
	B	134	100	103	88	696	539	0.764
	C	7	227	5	186	46	1189	0.774
Dog paces up and down, walks in circles, etc.	A	172	14	116	15	1230	100	0.275
	B	107	79	71	60	748	582	0.844
	C	5	181	2	129	49	1281	0.466
Dog pants frequently, even at rest	A	189	16	155	14	1173	99	0.961
	B	116	89	96	73	716	556	0.992
	C	11	194	12	157	35	1237	0.006
Dog shakes or trembles occasionally	A	499	46	124	10	898	73	0.809
	B	317	228	86	48	523	448	0.039 *
	C	18	527	8	126	32	939	0.278
Dog is lethargic	A	132	9	149	14	1236	104	0.796
	B	89	52	105	58	731	609	0.013 *
	C	7	134	4	159	46	1294	0.465
Dog is confident	A	1038	81	260	23	219	21	0.630
	B	642	477	153	130	132	108	0.529
	C	44	1075	7	276	6	234	0.379
Dog seems dull or depressed, not alert	A	32	3	83	4	1403	121	0.490
	B	16	19	57	30	853	671	0.101
	C	1	34	2	85	55	1469	0.914
Dog is anxious	A	502	43	277	22	736	63	0.977
	B	308	237	172	127	445	354	0.861
	C	12	533	14	285	32	767	0.090

Dog is considered to be very impulsive	A	421	30	303	27	789	70	0.611
	B	242	209	178	152	501	358	0.177
	C	14	437	14	316	30	829	0.684
Dog is curious	A	1361	113	103	11	46	4	0.671
	B	822	652	68	46	31	19	0.524
	C	54	1420	2	112	2	48	0.628
Dog is affectionate	A	1392	121	91	4	33	2	0.405
	B	846	667	56	39	23	12	0.454
	C	56	1457	1	94	1	34	0.411
Dog tends to be calm	A	890	76	291	29	334	22	0.365
	B	562	404	172	148	192	164	0.216
	C	32	934	7	313	19	337	0.086
Dog is aloof	A	251	24	218	15	1045	89	0.636
	B	162	113	134	99	629	505	0.550
	C	6	269	7	226	45	1089	0.351
Dog has more good days than bad days	A	1377	117	47	1	34	4	0.264
	B	840	654	27	21	26	12	0.344
	C	53	1441	1	47	3	35	0.233

Table S9. Questions by question types from the original survey [7].

Quesion type	Question
motor pattern	Dog buries toys / bones
	Dog enjoys playing with toys
	Dog howls
	Dog points
	Dog retrieves objects (e.g., balls, toys, sticks)
	Dog wants to play
	Dog Woo-woo barks
other behavior	Dog aggressively guards coveted items (e.g., stolen item, treats, food bowl)
	Dog appears to be sorry after HE has done something wrong
	Dog appears to have a lot of control over how HE responds
	Dog avoids other dogs
	Dog becomes aggressive when excited
	Dog behaves aggressively during visits to the veterinarian
	Dog behaves aggressively in response to perceived threats from people (e.g., being cornered, having collar reached for)
	Dog behaves aggressively toward other dogs
	Dog behaves aggressively towards cats
	Dog behaves aggressively towards unfamiliar people
	Dog behaves fearfully during visits to the veterinarian
	Dog behaves fearfully towards other dogs
	Dog behaves fearfully towards unfamiliar people
	Dog behaves fearfully when groomed (e.g., nails trimmed, brushed, bathed, ears cleaned)
	Dog behaves submissively (e.g., rolls over, avoids eye contact, licks his lips) when greeting other dogs
	Dog calms down very quickly after being excited
	Dog can be very persistent
	Dog chases bicycles, joggers, and skateboarders
	Dog damages doors, gates, or walls
	Dog does not think before he acts
	Dog doesn’t like to be approached or hugged
	Dog eats grass
	Dog eats his own feces
	Dog eats non-food items
	Dog enjoys life
	Dog escapes from cages, kennels, or enclosures
	Dog exhibits fearful behaviors when he is restrained
	Dog gets bored in play quickly
	Dog gets stuck behind objects and is unable to get around
	Dog has difficulty finding food dropped on the floor
	Dog has more good days than bad days
	Dog ignores commands
	Dog is a people person
	Dog is able to focus on a task in a distracting situation (e.g., loud or busy places, around other dogs)
	Dog is affectionate
	Dog is afraid of storms
	Dog is aloof
	Dog is anxious
	Dog is as active as he has been
	Dog is assertive or pushy with other dogs (e.g., if in a home with other dogs, when greeting)
	Dog is boisterous
	Dog is confident

Dog is considered to be very impulsive
Dog is curious
Dog is dominant over other dogs
Dog is easy to train
Dog is friendly towards other dogs
Dog is friendly towards unfamiliar people
Dog is highly sensitive to noise
Dog is lethargic
Dog is not keen to go into new situations
Dog is not very patient
Dog is playful with other dogs
Dog is quick to sneak out through open doors, gates
Dog is relaxed when greeting people
Dog is shy
Dog is slow to respond to corrections
Dog is very interested in and adapts easily to new things and new places
Dog kicks or scratches the ground after defecating
Dog knows he is a dog
Dog lays in one place all day long
Dog leaves food or objects alone when told to do so
Dog licks his empty bowl after finishing the food
Dog licks tile / linoleum floors
Dog likes to chase squirrels, birds, or other small animals
Dog marks with feces
Dog may become aggressive if he is frustrated with something
Dog moves normally
Dog must greet everyone who comes to the door
Dog paces up and down, walks in circles and/or wanders with no direction or purpose
Dog places his paw on my or other people's feet
Dog reacts very quickly
Dog responds to my presence
Dog seeks companionship from people
Dog seeks constant activity
Dog seems dull or depressed, not alert
Dog seems to get excited for no reason
Dog shows aggression when nervous or fearful
Dog shows barrier aggression
Dog shows extreme physical signs when excited
Dog shows handedness or side-preference
Dog shows submissive urination
Dog sleeps more, is less awake
Dog sometimes fails to recognize familiar people or pets
Dog stares blankly at the walls or floor
Dog takes a long time to lose interest in new things
Dog tends to be calm
Dog turns in circles before pooping
Dog walks away or avoids being patted
Dog whines to get attention, food, or toys
Dog whines when alone
Dog willingly shares his toys with other dogs
Dog works at tasks (e.g., getting treats out of a Kong, shredding toys) until entirely finished
Excitement can lead dog to fixed repetitive behavior

	When off leash, dog comes immediately when called
physical trait	Are dog's eyes different colors?<br>Does dog have soft fur or rough and bristly fur?<br>How long is dog's fur on his back and sides? Measure it against your finger.<br>How much white fur does dog have? Select the image with the closest amount of white.<br>Is dog's tail curly?<br>What color is dog? Select all that apply.<br>What is dog's ear shape? Select the image with the closest ear shape.<br>When dog is standing next to someone of average height, how high are his shoulders?
physical trait related	Dog avoids getting wet<br>Dog crosses his front paws<br>Dog drinks water quickly<br>Dog lifts his leg to urinate<br>Dog pants frequently, even at rest<br>Dog rests frog style<br>Dog shakes or trembles occasionally<br>Dog tilts his head

Figure S1. Dog breeds answered to be white (blue) and other colors (red) and the number of samples they appeared in. Only breeds appearing in more than one sample are presented.

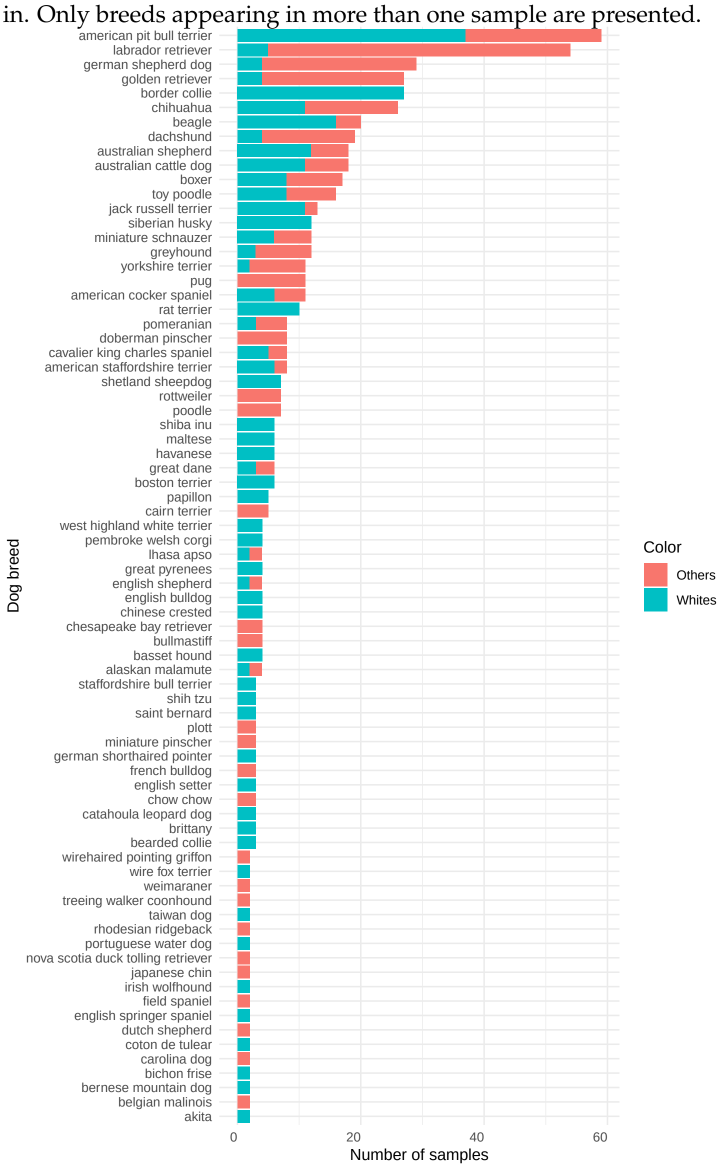

Figure S2. Dog breeds answered to be only white (blue) and not only white (red) and the number of samples they appeared in. Only breeds appearing in more than one sample are presented.

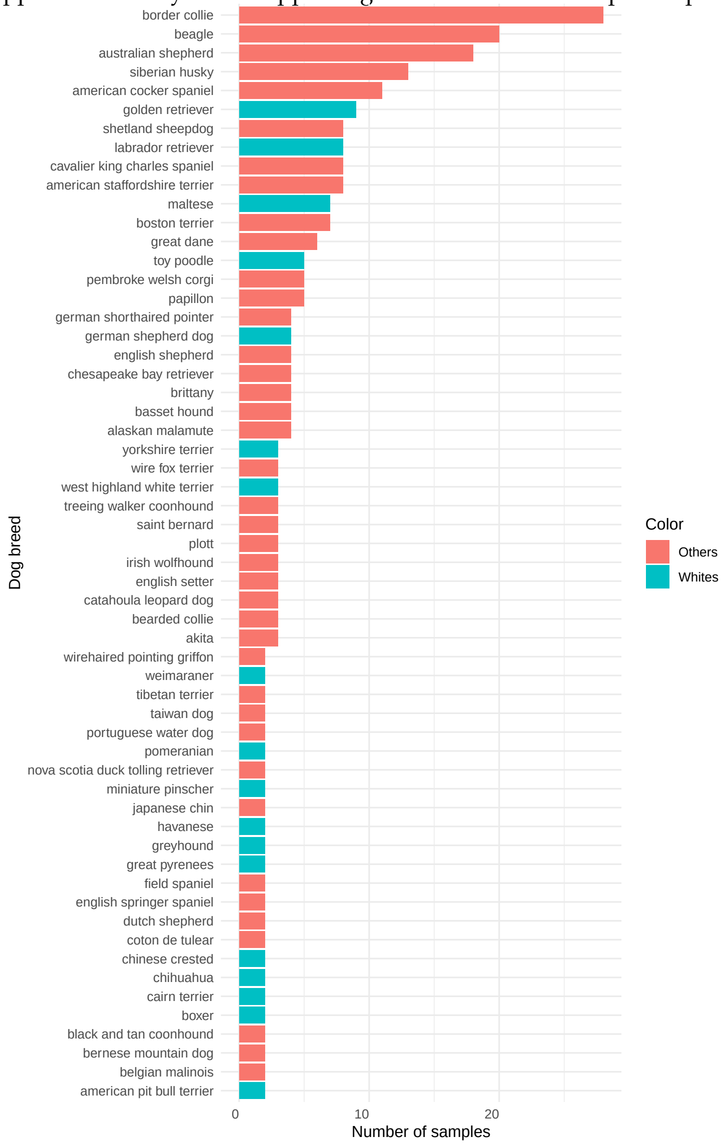

Supplement: Supplementary file 1 [file antibiotics-14-00433-s001.zip › antibiotics-3568611-SM.pdf]
