# Supplementary figures and images for: A One Health Approach Metagenomic Study on Antimicrobial Resistance Traits of Canine Saliva
Source: Antibiotics (Basel). 2025 Apr 25;14(5):433. doi: 10.3390/antibiotics14050433 (PMC12108403; doi:10.3390/antibiotics14050433)

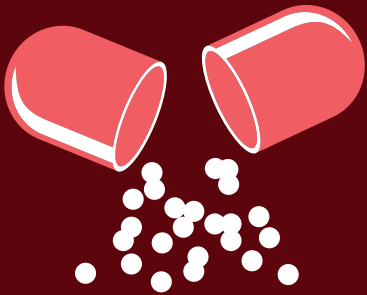

*antibiotics*

Supplement: Supplementary file 1 [file antibiotics-14-00433-s001.zip › Definitions/antibiotics-logo-eps-converted-to.pdf]

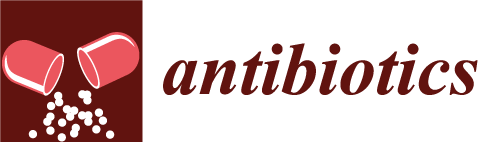

Supplement: Supplementary file 1 [file antibiotics-14-00433-s001.zip › Definitions/antibiotics-logo.png]

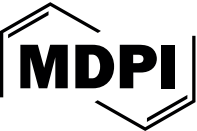

Supplement: Supplementary file 1 [file antibiotics-14-00433-s001.zip › Definitions/logo-mdpi-eps-converted-to.pdf]

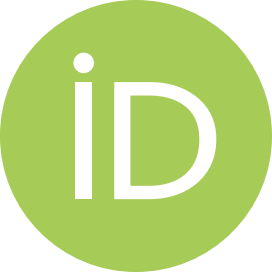

Supplement: Supplementary file 1 [file antibiotics-14-00433-s001.zip › Definitions/logo-orcid.pdf]

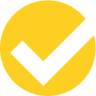

check for  
updates

Supplement: Supplementary file 1 [file antibiotics-14-00433-s001.zip › Definitions/logo-updates-eps-converted-to.pdf]

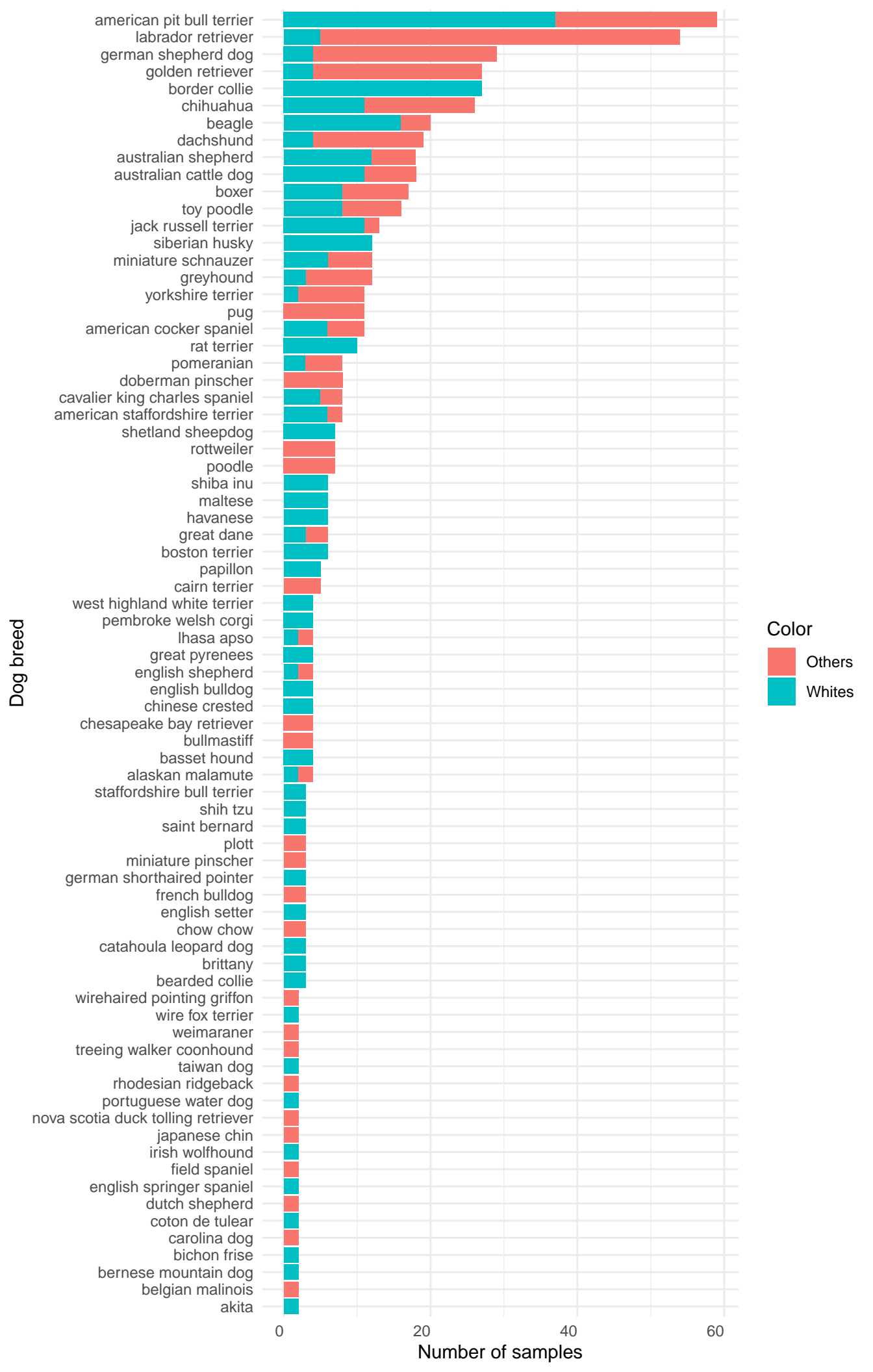

Supplement: Supplementary file 1 [file antibiotics-14-00433-s001.zip › wcolordogs1.pdf]

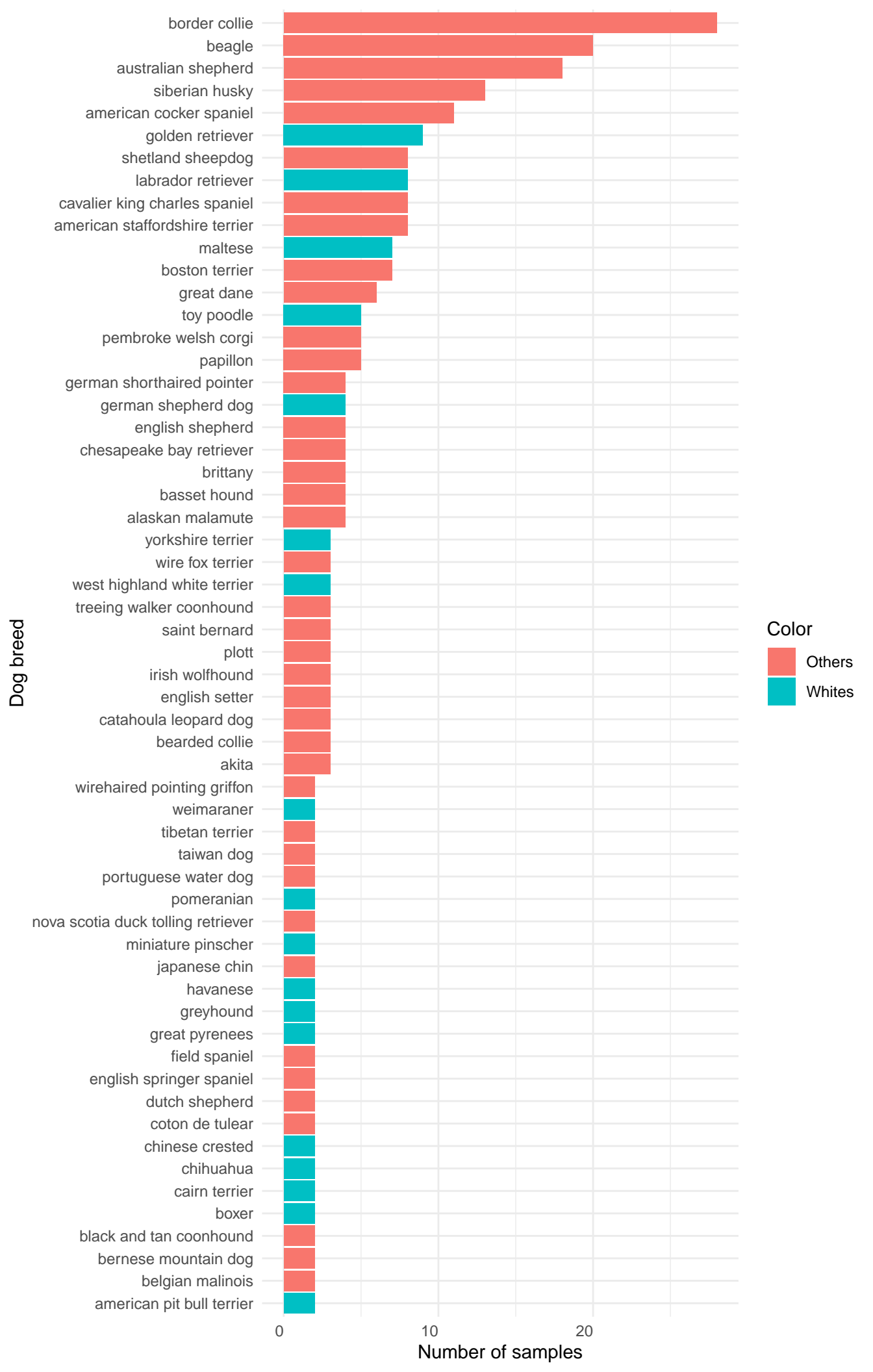

Supplement: Supplementary file 1 [file antibiotics-14-00433-s001.zip › wcolordogs2.pdf]
